# A non-invasive simplified scoring system to predict airway mucus plugs in asthma

**DOI:** 10.1186/s12890-026-04560-0

**Published:** 2026-08-04

**Authors:** Zhen Wang, Tiantian Xiong, Gongqi Chen, Chunli Huang, Wei Gu, Huiru Jie, Lixia Xia, Ruchong Chen, Yuchen Feng, Guohua Zhen

**Affiliations:** 1 Department of Respiratory and Critical Care Medicine, Tongji Hospital, Tongji Medical College, Huazhong University of Science and Technology, Wuhan, 430030 China; 2 National Health Commission Key Laboratory of Respiratory Diseases, Hubei Provincial Clinical Research Center for Chronic Airway Inflammatory Diseases, Wuhan, China; 3 Department of General Practice, Tongji Hospital, Tongji Medical College, Huazhong University of Science and Technology, Wuhan, China; 4 Key Laboratory of Respiratory Disease of Zhejiang Province, Department of Respiratory and Critical Care Medicine, Second Affiliated Hospital of Zhejiang University School of Medicine, Hangzhou, China; 5 State Key Laboratory of Respiratory Disease, National Center for Respiratory Medicine, Guangzhou Institute of Respiratory Health, The First Affiliated Hospital of Guangzhou Medical University, Guangzhou, China

**Keywords:** Asthma, Mucus plug, Severity, Clinical scoring system, Risk factors

## Abstract

**Background:**

Airway mucus plugs are a core pathological feature of severe asthma and are closely associated with airflow limitation and poor prognosis. However, rapid and efficient clinical screening tools for identifying airway mucus plugs in asthma are currently lacking.

**Objective:**

This study aimed to develop and evaluate meta‑analysis‑weighted scores for estimating the presence and severity of airway mucus plugs in adults with asthma.

**Methods:**

A systematic review and meta-analysis of six major databases (up to February 2025) were conducted to identify significant risk factors for mucus plugs. Subsequently, a mathematical equation-based score and a simplified scoring system were constructed based on the converted standardized effect-size weights (β). Internal validation of the scoring system was then performed using a cohort of 105 asthma patients from Tongji Hospital, Huazhong University of Science and Technology (2024∼2026).

**Results:**

The meta-analysis included 10 studies (837 patients) and identified age, pulmonary function parameters (FEV_1_%, FVC%, FEV_1_/FVC%), and type 2 inflammatory markers (FeNO, blood eosinophil count, IgE) as key associated factors. In the internal validation cohort, the simplified scoring system showed an AUC of 0.864 (95% CI: 0.785–0.943) for estimating mucus plug presence, with a specificity of 0.941 and sensitivity of 0.718. For estimating higher mucus plug burden, the AUC was 0.859 (95% CI: 0.777–0.941), with a sensitivity of 0.917 and specificity of 0.684.

**Conclusion:**

The meta-analysis-weighted scores showed promising preliminary discrimination for mucus plug presence and higher burden. These scores may assist in prioritizing patients for HRCT, though external validation is required.

**Supplementary Information:**

The online version contains supplementary material available at https://doi.org/10.1186/s12890-026-04560-0.

## Introduction

Asthma is a highly heterogeneous chronic inflammatory airway disease. Although severe asthma accounts for only a minority of the total patient population, it poses a significant challenge partly because many patients receive suboptimal therapy without endotyping, leading to poor disease control and a substantial healthcare burden [1]. In recent years, airway mucus plugs (MPs) have been identified as a critical pathological factor contributing to aggravated airflow limitation, accelerated decline in lung function, and disease exacerbations [2, 3]. Research indicates that extensive mucus plugging is present in up to 93% of fatal asthma cases [4]. Emerging biological evidence suggests that MPs are not merely passive secretory products but rather active pathogenic hubs—comprising mucins, eosinophils, and desquamated epithelial cells—that are closely linked to airway remodeling [5].

With the advancements in precision medicine, the identification of MPs has become increasingly vital for guiding Type 2 (T2) inflammation-targeted therapies (e.g., IL-5 inhibitors) and intensifying airway clearance strategies [6]. However, the detection of MPs currently relies primarily on high-resolution computed tomography (HRCT). Despite its high diagnostic accuracy, HRCT’s widespread application in long-term asthma management is severely constrained by the risks of ionizing radiation, high costs, and its impracticality as a tool for routine follow-up. Consequently, there is an urgent clinical need for a clinical screening tool based on easily accessible, non-invasive biomarkers to achieve early risk stratification and dynamic monitoring of MPs.

While previous studies have explored the associations between MPs and parameters such as age, lung function, and inflammatory markers [7–9], a standardized evidence-based clinical scoring system that integrates multi-dimensional clinical parameters remains lacking. In this study, we first conducted a systematic review and meta-analysis to comprehensively identify and quantify global risk factors associated with asthma-related MPs. Subsequently, based on the weighting coefficients derived from the meta-analysis, we constructed a mathematical equation-based score and a simplified clinical scoring system (the MP-Detect Score and MP-Severity Index), which were further validated in a retrospective cohort at our institution. This study aimed to provide clinicians with a practical, efficient, and non-invasive screening tool to optimize individualized diagnostic and therapeutic strategies for patients with asthma.

## Materials and methods

### Systematic review and meta-analysis

The meta-analysis was conducted and reported in accordance with the Preferred Reporting Items for Systematic Reviews and Meta-Analyses (PRISMA) statement. The study protocol was prospectively registered with the International Prospective Register of Systematic Reviews (PROSPERO) under the registration number CRD420251029254. We systematically searched electronic databases, including PubMed, Embase, the Cochrane Library, Web of Science, China National Knowledge Infrastructure (CNKI), and Wanfang databases, for relevant literature published from database inception through February 16, 2025. Detailed information regarding the literature screening process, inclusion/exclusion criteria, mucus plug scoring standards, data extraction, quality assessment, and statistical analysis is provided in Supplementary Data S1.

### Construction of the evidence-weighted scoring system based on meta-analysis results

#### Weight conversion 

We identified risk factors with statistical significance from the meta-analysis. Factors to be included in the scoring system were finalized after excluding overlapping variables with potential multicollinearity. Subsequently, we utilized the classic conversion formula proposed by Borenstein et al. [10] to convert the Standardized Mean Difference (SMD) into standardized effect-size weights (β): β = ln (OR); ≈ π/√3×SMD ≈ 1.81 × SMD. This approach was chosen to derive relative weights for each predictor based on aggregated evidence, rather than to estimate absolute log-odds for individual prediction, given the modest size of our internal validation cohort (*n* = 105).

#### Construction of the mathematical equation‑based score 

Given that the numerical scales of age, FeNO (ppb), and FEV_1_% differ significantly and cannot be directly aggregated, we normalized the raw data for each patient into Z-scores using the formula Z = (measured value - mean)/standard deviation, where the mean and standard deviation for each variable were derived from the internal validation cohort (*n* = 105). The complete list of normalization constants (means and standard deviations) is provided in Supplementary Table 2. By weighting these Z-scores with the standardized effect-size weights (β), we derived the Risk Score as follows: Risk Score = βage*Zage + β_FEV1%_*Z_FEV1%_ + …. This facilitated the development of the MP-Detect Score (Mucus Plug Detection Score) and the MP-Severity Index (Mucus Plug Severity Index).

#### Construction of the simplified scoring system 

To create a user-friendly clinical tool, the smallest β coefficient among the selected risk factors was defined as the baseline (1 point). The scores for other factors were then determined by dividing their respective β coefficients by the baseline β. To preserve relative weighting while maintaining clinical practicality, scores were rounded to the nearest 0.5-point increment, allowing half-point scores (e.g., 1.5, 3.5) for variables with intermediate weights. Finally, all risk factors were stratified and evaluated to establish the final scoring systems.

### Clinical validation

#### Study population

A retrospective cohort study was conducted at Tongji Hospital, Huazhong University of Science and Technology, from August 2024 to February 2026, for the purpose of internal validation of the scoring system. The inclusion criteria were: (1) age > 18 years; (2) diagnosis of asthma according to the Global Initiative for Asthma (GINA) guidelines; (3) availability of chest high-resolution computed tomography (HRCT) images; and (4) completion of pulmonary function tests, fractional exhaled nitric oxide (FeNO) measurement, and peripheral blood cell counts within one week of the HRCT scan. Exclusion criteria included acute respiratory infections, bronchiectasis, chronic obstructive pulmonary disease (COPD).

#### Assessment of airway mucus plugs

Chest HRCT images were independently evaluated by two physicians who were blinded to the clinical data. In cases of disagreement, a consensus was reached through discussion with a third senior radiologist. The presence and extent of mucus plugs were determined using a standardized 18-segment scoring system for the bronchial tree as previously described [11]. The upper respiratory tract, including the paranasal sinuses (PNS), was not assessed. Patients were categorized into the “Mucus Plug (MP) group” (score ≥ 1), the “High Mucus Plug group” (score ≥ 4), and the “Non-Mucus Plug (NMP) group” (score = 0).

#### Statistical analysis of the internal validation cohort

Data analysis for the clinical internal validation cohort was performed using SPSS version 26.0 and R software (version 4.3.1). Normally distributed continuous variables are expressed as mean ± standard deviation (x ± s), with intergroup comparisons conducted using independent samples t-tests. Non-normally distributed data are presented as median (interquartile range [IQR]) and compared using the Mann-Whitney U test. Categorical variables are expressed as frequencies (percentages) and analyzed using the X^2^ test or Fisher’s exact test.

The discriminatory power of the scoring system was evaluated using Receiver Operating Characteristic (ROC) curves, with the calculation of the Area Under the Curve (AUC) and its 95% confidence interval (CI). The optimal cut-off values were determined using Youden’s Index, alongside the calculation of sensitivity and specificity. Decision Curve Analysis (DCA) was employed to assess the clinical net benefit of the scoring system across various threshold probabilities. Scoring system calibration was assessed using the Brier score, which quantifies the overall accuracy of predicted probabilities, with values closer to 0 indicating better calibration. To assess scoring system stability and potential overfitting, we performed 10‑fold cross‑validation and bootstrap internal validation with 500 resamples. The optimism‑corrected area under the curve (AUC) and 95% confidence intervals were calculated from the bootstrap resamples.

To evaluate the diagnostic performance of the simplified scoring system relative to the mathematical equation‑based score, the DeLong test was used for head-to-head comparisons of their respective AUCs. A P-value > 0.05 indicated that the simplified scoring system maintained discriminative performance similar to the mathematical equation‑based score while offering greater clinical practicality. Statistical significance was defined as *P* < 0.05.

To evaluate the impact of multicollinearity among the pulmonary function indices (FEV_1_%, FVC%, and FEV_1_/FVC%), collinearity diagnostics were performed by calculating variance inflation factors (VIFs). Additionally, a sensitivity analysis was conducted by constructing a restricted mathematical scoring system that excluded FEV_1_% and FVC% (retaining only FEV_1_/FVC%, which had the highest beta coefficient) to examine the changes in AUC, sensitivity, and specificity in the internal validation cohort. This study was conducted in accordance with the TRIPOD + AI statement [12]. (Supplementary S2-TRIPOD Checklist).

## Results

### Systematic review and meta-analysis

#### Characteristics of included studies

A total of 3,055 records were initially retrieved from electronic databases (PubMed: 1,466; Embase: 1,185; Cochrane Library: 57; Web of Science:79; CNKI: 47; Wanfang Data: 221). Based on the predefined inclusion and exclusion criteria, 10 studies encompassing a total of 837 patients were ultimately included in the analysis. Among these, one was a randomized controlled trial (RCT), one was a cross-sectional study, and eight were cohort studies. The baseline characteristics of the included studies are summarized in Table 1.

**Table 1:** Characteristics of the included studies

Author, Year	Research type	Country	Sample size	Research indicators
Tang. 2022 [7]	Cohort study	America	126	1–2、4–15
Mummy. 2022 [8]	Cohort study	Wisconsin, America	44	1–2、4–13
Chan. 2023 [9]	Cohort study	England	126	6–8、10、13–15
Tamura. 2022 [13]	Cohort study	Japan	49	1–11、14
Nordenmark. 2023 [14]	RCT	Europe and North America	91	1–2、4、6、10、13–15
Liu. 2023 [15]	Cross-sectional study	Beijing China	100	1–8、12、13–15
Yang. 2024 [16]	Cohort study	Shandong China	120	1、3–4
Dunican 2018 [11]	Cohort study	America	85	1–2、4、6、13
Audousset 2024 [17]	Cohort study	Canada	38	1–2、4、6、10、13
Götschke 2025 [18]	Cohort study	Germany	58	1–2、4、6、10、13

Research indicators: (1) Gender (female); (2) Age; (3) Smoking history; (4) BMI; (5) Diagnosis of severe asthma; (6) FEV_1_%; (7) FVC%; (8) FEV_1_/FVC%; (9) Sputum eosinophils count%; (10) Peripheral blood eosinophils count; 11. Sputum neutrophil count%; 12. Peripheral blood neutrophil count; 13. FeNO; 14. IgE; 15. Number of visits for acute exacerbation of asthma

#### Risk factors

**Fig. 1:**
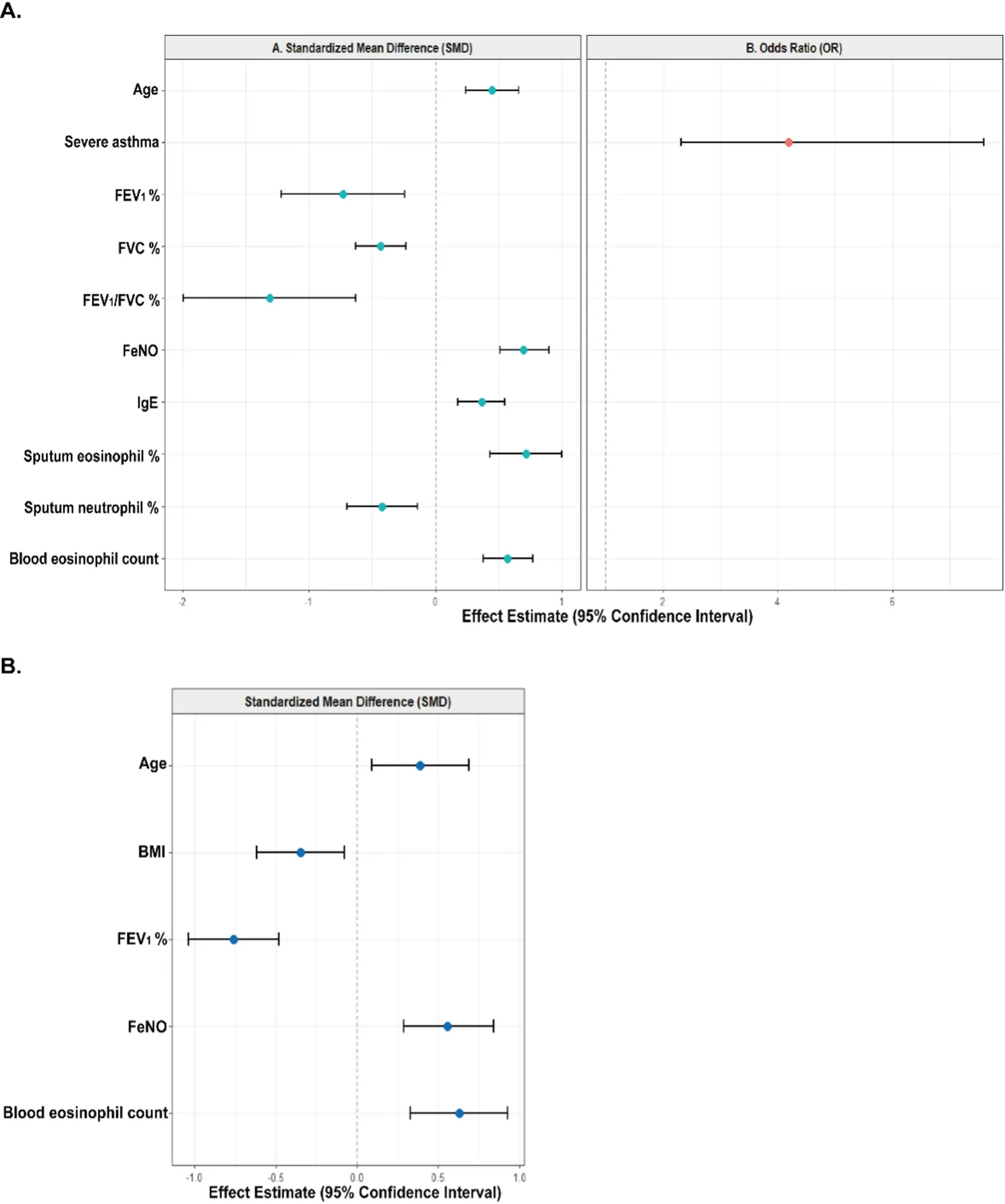
A meta-analysis of risk factors for mucus plugs in asthma. **A** Meta analysis of the clinical characteristics of asthma patients with and without mucus plug. B Meta analysis of the clinical characteristics of asthma patients with high and low mucus plug scores.

A total of 15 risk factors were evaluated in the meta-analysis. By comparing asthma patients with and without mucus plugs, we found that patients with MPs were significantly older and predominantly presented with severe asthma. These patients exhibited more pronounced airflow limitation, characterized by significantly lower FEV_1_%, FVC%, and FEV_1_/FVC%. Furthermore, regarding airway inflammation, patients with MPs demonstrated significantly higher levels of FeNO, peripheral blood eosinophil counts, and sputum eosinophil percentages. In contrast, sputum neutrophil percentage was significantly lower in the MP group compared to the NMP group (Fig. 1A).

Additionally, we assessed the clinical impact of mucus plug severity. The results indicated that higher MP scores were associated with advanced age, lower BMI, reduced FEV_1_%, and increased peripheral blood eosinophilia (Fig. 1B). For the variable of acute exacerbations (indicator 15 in Table 1), definitions varied across studies; detailed definitions and subgroup analyses are provided in Supplementary Data S1, Comparison between mathematical equation and simplified scoring system. Detailed results of the meta-analysis, including heterogeneity tests and sensitivity analyses for each risk factor, are provided in Supplementary Data S1.

### Development of the scoring systems

#### Construction of the mathematical equation‑based score based on meta-analysis weights

Through the meta-analysis comparing patients with and without mucus plugs, we identified 10 statistically significant risk factors: age (SMD = 0.45, 95% CI: 0.24∼0.66, *P* < 0.001), severe asthma (OR = 4.19, 95% CI: 2.32∼7.59, *P* < 0.0001), FEV_1_% (SMD = -0.73, 95% CI: -1.22∼-0.24, *P* = 0.004), FVC% (SMD = -0.43, 95% CI: -0.63∼-0.23, *P* < 0.001), FEV_1_/FVC% (SMD = -1.31, 95% CI = -1.99∼-0.63, *P* < 0.001), FeNO (SMD = 0.70, 95% CI: 0.51∼0.90, *P* < 0.001)、sputum eosinophil percentage (SMD = 0.72, 95% CI: 0.43∼1.00, *P* < 0.001), sputum neutrophil percentage (SMD = -0.42, 95% CI: -0.70∼-0.14, *P* = 0.004), peripheral blood eosinophil count (SMD = 0.57, 95% CI: 0.38∼0.77, *P* < 0.001), and IgE (SMD = 0.37, 95% CI: 0.18∼0.55, *P* < 0.0001).

Regarding the severity of mucus plugs (high vs. low scores), the meta-analysis identified five significant risk factors: age (SMD = 0.39, 95% CI: 0.09∼0.69, *P* = 0.01), FeNO (SMD = 0.56, 95% CI: 0.29∼0.84, *P* < 0.001), peripheral blood eosinophil count (SMD = 0.63, 95% CI: 0.33∼0.93, *P* < 0.001), BMI (SMD = -0.35, 95% CI: -0.62∼-0.08, *P* = 0.01), and FEV_1_% (SMD = -0.76, 95% CI: -1.04∼-0.48, *P* < 0.001).

Considering the simplicity and non-invasiveness in clinical practical application, seven of the most representative non-invasive indicators were selected in the internal validation cohort of this study. To quantitatively evaluate these factors, the Standardized Mean Differences (SMDs) derived from the meta-analysis were converted into standardized effect-size weights (β). To eliminate dimensional differences across various clinical indicators, Z-score normalization was applied to the variables. This process culminated in the construction of a continuous variable mathematical equation-based score (Table 2).

**Table 2:** Standardized effect‑size weights (β) derived from meta‑analysis for the construction of mathematical equation‑based scores for mucus plugs

Risk factors	Meta combined effect size (95% CI)	β	Score Ratio	Final Points
MP-Detect Score				
FEV₁/FVC%	SMD = -1.31	-2.38	3.54	3.5
FEV₁%	SMD = -0.73	-1.32	1.97	2.0
FeNO	SMD = 0.70	1.27	1.89	2.0
blood eosinophil count	SMD = 0.57	1.03	1.54	1.5
Age	SMD = 0.45	0.82	1.21	1.0
FVC%	SMD = -0.43	-0.78	1.16	1.0
IgE	SMD = 0.37	0.67	1.00 (standard)	1.0
MP-Severity Index				
Age	SMD = 0.39	0.71	1.11	1
FeNO	SMD = 0.56	1.02	1.44	1.5
blood eosinophil count	SMD = 0.63	1.14	1.61	1.5
FEV₁%	SMD = -0.76	-1.38	1.95	2
BMI	SMD = -0.35	-0.64	1.00 (standard)	1

MP-DetectScore = 0.82*Zage + (-1.32) *Z_FEV1%_ + (-2.38) *Z_FEV₁/FVC%_ + 1.27*Z_FeNO_ + 1.03*Z_blood eosinophil count_ + (-0.78) *Z_FVC%_+0.67*Z_IgE_; MP-SeverityIndex = 0.71*Zage + 1.02*Z_FeNO_ + 1.14*Z_blood eosinophil count_+(-1.38) *Z_FEV₁%_ + (-0.35) *Z_BMI_

#### Construction of a simplified clinical scoring system

Recognizing the complexity of utilizing mathematical equations for rapid bedside assessment, we further developed a simplified scoring system based on risk stratification. This scoring system converts continuous variables into categorical variables and assigns points based on the proportion of the standardized effect‑size weights (β). The cutoffs for each variable were selected according to the following principles. For pulmonary function parameters (FEV₁%, FEV₁/FVC%, and FVC%), we used widely accepted clinical thresholds (80% and 60% for FEV₁%; 70% and 60% for FEV₁/FVC%; 80% for FVC%) consistent with GINA guidelines, as these values are routinely recognized by clinicians in daily practice. For the remaining variables—including age, FeNO, blood eosinophil count, IgE, and BMI—cutoffs were determined by the optimal Youden index derived from receiver operating characteristic (ROC) curves in our internal validation cohort (detailed in Supplementary Table 1). To enable clinicians to better identify the high-risk features of mucus plugs observed in the data, lung function parameters were classified based on internationally recognized thresholds, and FEV₁% and FEV₁/FVC% were stratified into a three-level scoring system.

In the MP-Detect Score, an FEV_1_/FVC% ≤ 60% was assigned the highest score (3.5 points), followed by an FEV_1_% ≤ 60% (2 points) and FeNO ≥ 35 ppb (2 points). In the MP-Severity Index, an FEV_1_% ≤ 60% and indicators of high Type 2 (T2) inflammation (e.g. FeNO ≥ 35 ppb or peripheral blood eosinophil count ≥ 0.35 × 10⁹/L) served as the primary weighted components for estimating severe mucus plugging (Table 3).

**Table 3:** Simplified scoring systems for airway mucus plugs based on standardized effect‑size weights (β) from the meta-analysis

Risk factors	Risk stratification	Points
MP-Detect Score		
FEV₁/FVC%	≤ 60%	3.5
	> 60% to ≤ 70%	1.5
	>70%	0
FEV₁%	≤ 60%	2
	> 60% to ≤ 80%	1
	> 80%	0
FeNO (ppb)	≥ 35	2
	< 35	0
blood eosinophil count (*10^9/L)	≥ 0.35	1.5
	< 0.35	0
Age	≥ 52.00	1
	< 52.00, > 18	0
FVC%	≤ 80%	1
	> 80%	0
IgE (IU/mL)	≥ 611.40	1
	< 611.40	0
MP-Severity Index		
Age	≥ 52	1
	< 52, > 18	0
FeNO (ppb)	≥ 35	1.5
	< 35	0
blood eosinophil count(*10^9/L)	≥ 0.35	1.5
	< 0.35	0
FEV₁%	≤ 60%	2
	> 60% to ≤ 80%	1
	> 80%	0
BMI (kg/m2)	≤ 19.53	1
	> 19.53	0

### Clinical cohort for score derivation and internal validation

#### Baseline characteristics of the study cohort

A total of 105 asthma patients were included in the validation cohort, of whom 71 (67.6%) presented with airway mucus plugs (MP group) and 34 (32.4%) did not (NMP group). For MP‑Severity, patients with MP scores ≥ 4 were classified as the high‑score group (*n* = 48), and those with MP scores < 4 as the low‑score group (*n* = 57). As summarized in Table 4, patients in the MP group were significantly older than those in the NMP group (56.13 ± 10.38 vs. 42.56 ± 7.92, *P* < 0.001).

**Table 4:** Baseline characteristics of the validation cohort stratified by mucus plug presence

Characteristic	Total (*N* = 105)	MP Group (*n* = 71)	NMP Group (*n* = 34)	*P* value
Demographics				
Age, years	51.73 ± 11.48	56.13 ± 10.38	42.56 ± 7.92	**< **0.001
Sex (Male/Female)	41/64	27/44	14/20	0.924
BMI, kg/m²	23.89 ± 3.67	23.80 ± 3.39	24.08 ± 4.30	0.716
Lung Function				
FEV_1_(% predicted)	75.29 ± 24.20	68.29 ± 25.18	89.90 ± 13.90	**<** 0.001
FVC (% predicted)	99.66 ± 19.76	96.06 ± 20.76	107.18 ± 15.54	0.007
FEV_1_/FVC ratio (%)	61.74 ± 12.83	57.60 ± 12.88	70.37 ± 7.61	**< **0.001
Inflammatory Markers				
FeNO, ppb	24.5 (18.0–52.0)	36.0 (19.0–52.0)	27.5 (17.0–56.0)	0.557
Blood Eos, ×10^9^/L	0.24 (0.10–0.53)	0.36 (0.14–0.81)	0.15 (0.07–0.24)	**< **0.001
Total IgE, IU/mL	223.2 (78.8–597.3)	225.1 (94.9–637.3)	208.0 (66.4–499.0)	0.410

Regarding pulmonary function, the MP group exhibited more severe airflow limitation and restrictive ventilatory impairment. Specifically, the percent-predicted forced vital capacity (FVC%) (96.06 ± 20.76 vs. 107.18 ± 15.54, *P* = 0.007), percent-predicted forced expiratory volume in 1 s (FEV_1_%) (68.29 ± 25.18 vs. 89.90 ± 13.90, *P* < 0.001), and the FEV_1_/FVC% (57.60 ± 12.88% vs. 70.37 ± 7.61%, *P* < 0.001).

Although FeNO levels (median: 36.0 [IQR: 19.0–52.0] vs. 27.5 [IQR: 17.0–56.0] ppb) and total IgE levels (median: 225.1 [IQR: 94.9–637.3] vs. 208.0 [IQR: 66.4–499.0] IU/mL) were numerically higher in the MP group, these differences did not reach statistical significance (*P* = 0.557 and *P* = 0.410, respectively). Peripheral blood eosinophil counts were significantly elevated in the MP group (median: 0.36 [IQR: 0.14–0.81] vs. 0.15 [IQR: 0.07–0.24] × 10⁹/L, *P* < 0.001). No significant differences were observed between the two groups regarding sex distribution or BMI.

#### Diagnostic performance of the mathematical equation‑based score

**Fig. 2:**
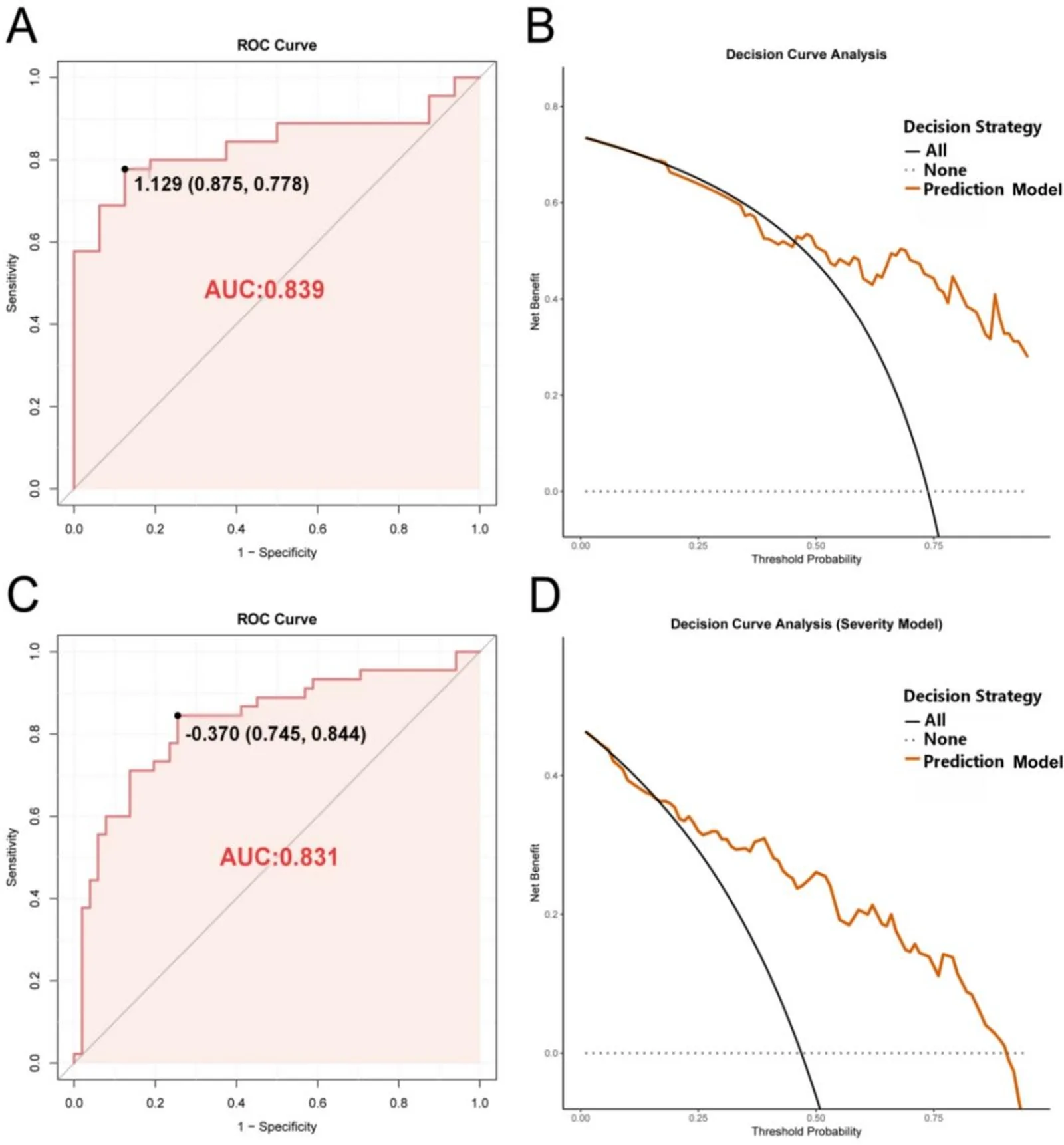
Diagnostic performance of the mathematical equation equation‑based score for airway mucus plugs in asthma. **A** ROC curve for estimating the presence of MPs (MP-Detect, AUC: 0.839). The optimal cutoff was 1.129 (specificity: 0.875, sensitivity: 0.778). **C** ROC curve for assessing MP severity (MP-Severity, AUC: 0.831). The optimal cutoff was -0.370 (specificity: 0.745, sensitivity: 0.844). **B**, **D** Net benefit decision curves for MP-Detect and MP-Severity, respectively (black solid line: treat all; dashed line: treat none; yellow solid line: prediction model)

The Z-score-based mathematical equation‑based score demonstrated robust discriminative ability in identifying both the presence and severity of airway mucus plugs. For estimating the presence of MPs (MP-Detect), the Area Under the Receiver Operating Characteristic Curve (AUC) reached 0.839 (95% CI: 0.755∼0.923) (Fig. 2A). At the optimal cut-off value of 1.129, the score yielded a sensitivity of 0.778 and a high specificity of 0.875. For assessing MP severity (MP-Severity), the AUC was 0.831 (95% CI: 0.741∼0.921) (Fig. 2C), with a sensitivity of 0.844 and a specificity of 0.745 at a cut-off value of -0.370. Decision Curve Analysis (DCA) demonstrated that across clinically relevant threshold ranges (approximately 0.1 to 0.9), the mathematical equation‑based score provided a higher net benefit compared with both ‘treat all‘ and ‘treat none‘ strategies, supporting its potential clinical utility (Fig. 2B, D). The Brier scores for the mathematical equation‑based score were 0.142 for MP-Detect and 0.165 for MP-Severity, indicating good calibration.

Internal validation of the mathematical equation‑based score demonstrated good stability and calibration. 10‑fold cross‑validation yielded mean AUCs of 0.825 ± 0.075 for MP‑Detect and 0.818 ± 0.084 for MP‑Severity. Bootstrap validation with 500 resamples showed minimal overfitting, with optimism‑corrected AUCs of 0.822 (95% CI: 0.755–0.923) for MP‑Detect and 0.814 (95% CI: 0.741–0.921) for MP‑Severity, corresponding to optimism values of 0.017 for both scores. Calibration was satisfactory, with Brier scores of 0.153 and 0.119, and calibration slopes of 1.039 and 0.971, respectively (Supplementary Table 3).

#### Diagnostic performance of the simplified scoring system

**Fig. 3:**
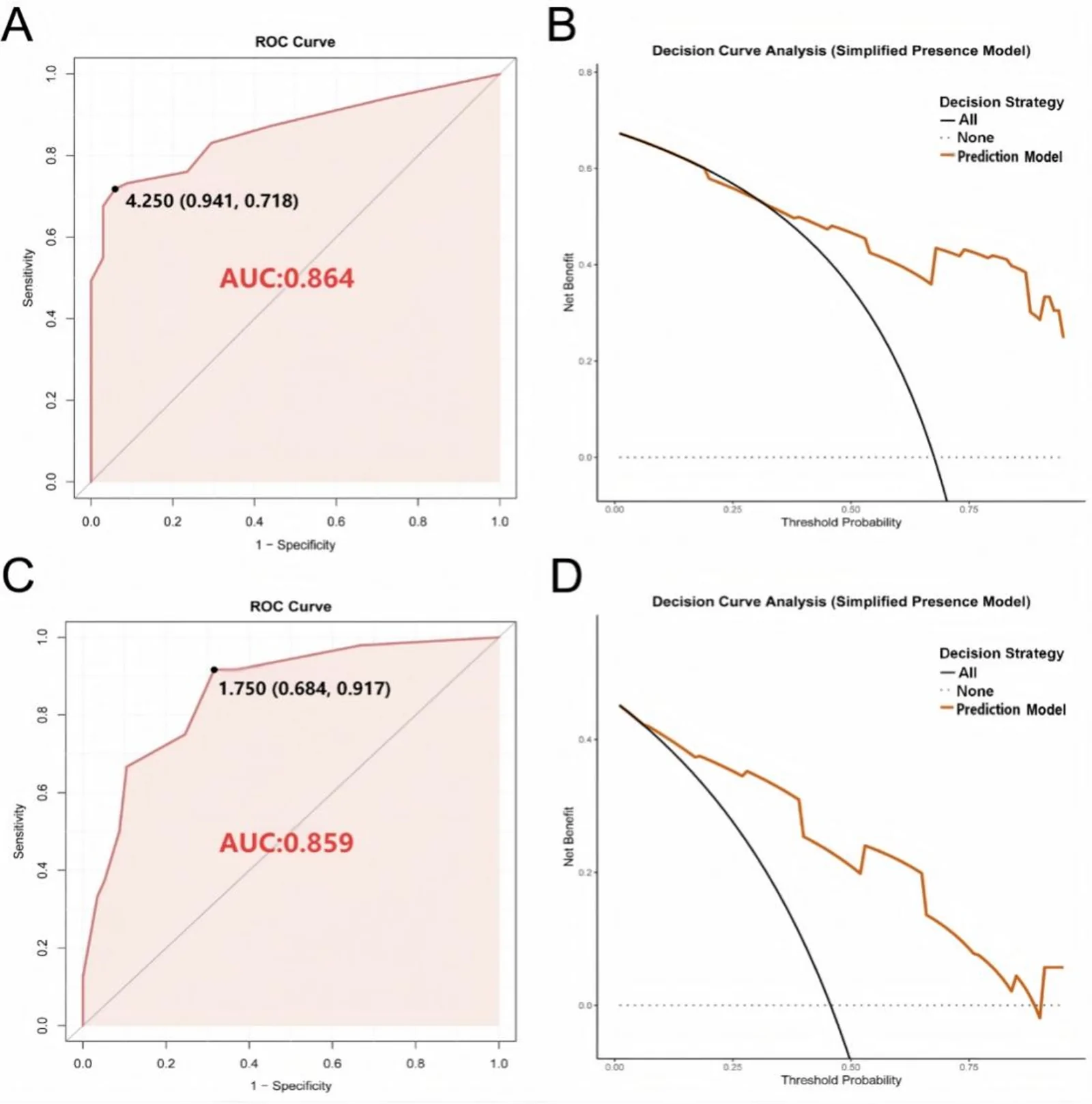
Diagnostic performance of the simplified scoring system for airway mucus plugs in asthma. **A** ROC curve for estimating the presence of MPs (simplified MP-Detect , AUC: 0.864). The optimal cutoff was 4.250 (specificity: 0.941, sensitivity: 0.718). **C** ROC curve for assessing MP severity (simplified MP-Severity, AUC: 0.859). The optimal cutoff was 1.750 (specificity: 0.684, sensitivity: 0.917). **B**, **D** Net benefit decision curves for simplified MP-Detect and simplified MP-Severity, respectively (black solid line: treat all; dashed line: treat none; yellow solid line: prediction model)

To facilitate clinical implementation, the simplified scoring system was evaluated. The AUC for estimating MP presence (MP-Detect) reached 0.864 (95% CI: 0.785∼0.943) (Fig. 3A). Using 4.250 as the cut-off value, the scoring system exhibited excellent specificity (0.941) and sensitivity (0.718). For the assessment of MP severity (MP-Severity), the AUC was 0.859 (95% CI: 0.777∼0.941) (Fig. 3C). At a threshold of 1.750, the sensitivity was as high as 0.917, ensuring an extremely low rate of missed diagnosis for patients with severe mucus plugging. The DCA curves further confirmed that the simplified scoring system provided a net benefit across clinically relevant threshold ranges (approximately 0.1 to 0.9), suggesting its potential value as a practical rapid screening tool in resource‑limited settings (Fig. 3B, D). The Brier scores for the simplified scoring system were 0.141 for MP-Detect and 0.151 for MP-Severity, indicating good calibration.

Internal validation demonstrated good stability of the scoring system. 10‑fold cross‑validation yielded mean AUCs of 0.846 ± 0.069 for MP‑Detect and 0.842 ± 0.088 for MP‑Severity. Bootstrap validation with 500 resamples showed minimal overfitting, with optimism‑corrected AUCs of 0.847 (95% CI: 0.768–0.926) for MP‑Detect and 0.841 (95% CI: 0.759–0.923) for MP‑Severity, corresponding to optimism values of 0.017 and 0.018, respectively (Supplementary Table 3).

### Comparison between mathematical equation and simplified scoring system

The performance of the simplified scoring system was compared directly with the mathematical equation-based score. Regarding the estimation of MP presence, a DeLong test was performed to compare the discriminative power of the two approaches. The results showed no statistically significant difference between the simplified scoring system and the mathematical equation-based score (Z = 1.15, *P* = 0.25). For estimating MP severity, no statistically significant difference was observed between the two approaches (Z = 0.98, *P* = 0.32). Notably, the simplified scoring system showed a higher sensitivity (0.917) at the cut-off value of 1.750, enabling more effective identification of patients with severe mucus plugs, while offering greater ease of use for clinical application (Table 5).

**Table 5:** Comparison of performance between the mathematical equation‑based score and simplified scoring system

	Score type	AUC (95% CI)	Sensitivity	Specificity	Z	*P*
MP-Detect	Mathematical equation‑based score	0.839 (0.755–0.923)	0.778	0.875	-	-
	Simplified scoring system	0.864 (0.785–0.943)	0.718	0.941	1.15	0.25
MP-Severity	Mathematical equation‑based score	0.831 (0.741–0.921)	0.844	0.745	-	-
	Simplified scoring system	0.859 (0.777–0.941)	0.917	0.684	0.98	0.32

### Comparison with multivariable logistic regression

To benchmark our evidence‑based scoring system against a traditional regression‑based approach, we constructed multivariable logistic regression models in the validation cohort. For MP‑Detect (predictors: age, FEV₁%, FVC%, FEV₁/FVC%, FeNO, blood eosinophil count, and IgE), the model achieved an AUC of 0.852 (95% CI: 0.752–0.918), which was not statistically different from our simplified scoring system (AUC = 0.864, 95% CI: 0.785–0.943). For MP‑Severity (predictors: age, BMI, FEV₁%, FeNO, and blood eosinophil count), the multivariable logistic regression achieved an AUC of 0.932 (95% CI: 0.864–0.999), compared with 0.859 (95% CI: 0.777–0.941) for the simplified scoring system. The DeLong test confirmed that there was no statistically significant difference in discriminative performance between the simplified scoring system and the multivariable logistic regression approach for either MP‑Detect (Z = 0.78, *P* = 0.436) or MP‑Severity (Z = 1.341, *P* = 0.180) (Supplementary Table 4). However, a non‑significant DeLong test does not establish equivalence; the primary advantage of the simplified scoring system lies in its ease of clinical application rather than superior diagnostic accuracy.

Furthermore, collinearity diagnostics in the validation cohort revealed that the VIFs for FEV₁%, FVC%, and FEV₁/FVC% were 12.86, 7.95, and 6.35, respectively (Supplementary Table 5). The VIF for FEV₁% exceeded the conventional threshold of 10, confirming statistical collinearity. In the sensitivity analysis, the restricted mathematical system (retaining only FEV₁/FVC% among the lung function variables) yielded an AUC of 0.812 (95% CI: 0.721–0.903), with a sensitivity of 74.6% and a specificity of 82.4%. Similarly, alternative restricted scoring system retaining only FEV₁% or FVC% also failed to improve the AUC (yielding AUCs of 0.822 and 0.803, respectively; Supplementary Table 6). Both discrimination (AUC dropped from 0.839 to 0.812) and clinical safety metrics (sensitivity dropped from 77.8% to 74.6%) were compromised in the restricted model compared with the original comprehensive mathematical score, highlighting the additive diagnostic value of the excluded pulmonary function parameters (Supplementary Table 5).

## Discussion

This study describes the development and internal validation of a non‑invasive evidence‑weighted clinical scoring system aimed at identifying airway mucous plugs (MPs) and assessing their severity in asthma patients. By integrating data from 10 studies encompassing 837 patients, our meta-analysis robustly confirmed that older age, severe airflow limitation (low FEV_1_% predicted), and pronounced type 2 inflammation (elevated FeNO and blood eosinophil counts) are closely associated with MP formation. Based on these findings, we developed the MP-Detect Score and MP-Severity Index, which demonstrated discriminative ability for identifying MPs, with the simplified scoring system achieving AUCs of 0.864 and 0.859, respectively. Our simplified scoring system provides a tool for assessing the risk of airway lumen obstruction in asthma.

MP formation is a central pathophysiological event in severe asthma [19]. Our results further elucidate the tight coupling between type 2 inflammation and mechanical obstruction. The meta-analysis findings robustly support high peripheral blood eosinophil counts, FeNO, and IgE as significant risk factors associated with MP presence. Eosinophils are key effector cells in type 2 inflammation, releasing inflammatory mediators such as major basic protein (MBP) and eosinophil peroxidase (EPO) that promote excessive mucus secretion [20–22]. Concurrently, type 2 inflammation stimulates goblet cell hyperplasia and high expression of mucin 5AC (MUC5AC), further increasing mucus production [23, 24]. In a study by Han et al. [25], among children with acute asthma exacerbation, those with airway mucus plugs exhibited higher serum total IgE levels and were more prone to hospitalization. These findings indicate that type 2 inflammation plays a pivotal role in the formation of airway mucus plugs. Recently, Sasada et al. [26] developed the ABENF score, a practical clinical tool for estimating CT‑detected mucus plug presence using age, BMI, blood eosinophil count, FeNO, and FEV_1_%. While the ABENF score demonstrated good discrimination (AUC: 0.781–0.836) in multicenter cohorts, our study differs in two aspects: (1) predictor weights were derived from meta‑analysis pooled effect sizes rather than from a single cohort; and (2) we additionally developed a severity index (MP‑Severity Index) beyond a binary detection score. No head‑to‑head comparison was performed, so no conclusions regarding superiority can be drawn.

Our scoring system transforms this “inflammation-function” axis into a quantitative risk score. Although FeNO and IgE did not reach statistical significance (*P* > 0.05) in our validation cohort, this may be attributable to sample size limitations or the suppressive effects of prior inhaled corticosteroid (ICS) use, leading to underestimated values. Nevertheless, retaining these variables in score construction respects the robust evidence from large-sample meta-analyses and ensures the scoring system’s validity across diverse clinical contexts. Furthermore, both our meta-analysis and cohort study indicated that older patients are more prone to MP formation. This may be due to immunosenescence and age-related decline in mucociliary clearance, hindering mucus expulsion [27]. Concurrently, elderly asthma patients often present with a more chronic form of the disease, with a progressively weakened immune system and diminished capacity for regulating inflammation, thereby exacerbating airway inflammation and MP formation [28, 29].

Pulmonary function parameters, particularly the FEV_1_/FVC% and FEV_1_%, carried the highest weight in the scoring systems, aligning perfectly with the physical airway obstruction caused by MPs in large and medium-sized airways [8, 9, 11, 13]. In the simplified scoring system, an FEV_1_/FVC% ≤ 60% was assigned a high weight of 3.5 points, reflecting that severe airflow limitation is a sensitive marker for MPs. Our validation data further confirmed that patients in the MP group exhibited significantly more severe ventilatory dysfunction than those in the non-MP group, with a mean FEV_1_% predicted of only 68.29 ± 25.18. This association between functional impairment and pathological structure suggests that in patients with persistently poor lung function who respond inadequately to conventional ICS therapy, clinicians should maintain a high index of suspicion for “silent” airway obstruction due to MPs, rather than solely attributing it to bronchospasm. Therefore, early identification of asthma patients with airway mucus plugs and proactive intervention targeting airflow obstruction and eosinophilic inflammation to reduce airway mucus plug formation are of paramount importance. Biologics such as anti-TSLP (tezepelumab), anti-IL-5/IL-5R (e.g., mepolizumab, benralizumab, reslizumab), and anti-IL-4Rα (dupilumab) can target upstream type 2 inflammatory pathways (ILC2 activation, eosinophilopoiesis, IgE synthesis, epithelial alarmin release) [30], which are intrinsically linked to mucus hypersecretion and plug formation [31–33]. Recent reviews have further framed mucus plugging as a measurable and potentially modifiable treatable trait in asthma and other chronic airway diseases [34, 35]. Within this framework, mucus abnormalities represent a distinct phenotype that supports personalized management strategies, particularly in the context of type 2‑targeted biologic therapies and quantitative CT imaging [34, 35]. Our MP‑Detect Score and MP‑Severity Index align with this concept by providing a non‑invasive tool to identify patients with significant mucus burden, who may be candidates for more intensive anti‑inflammatory or mucoactive interventions.

Collinearity diagnostics in our validation cohort yielded Variance Inflation Factors (VIFs) of 12.86 for FEV₁%, 7.95 for FVC%, and 6.35 for FEV₁/FVC%, respectively. However, sensitivity analysis demonstrated that constructing a restricted model excluding FEV_1_% and FVC% compromised diagnostic performance, with the mathematical equation‑based score’s AUC dropping from 0.839 to 0.812, sensitivity from 77.8% to 74.6%, and specificity from 87.5% to 82.4%. Similarly, alternative restricted scoring system retaining only FEV₁% or FVC% also failed to improve diagnostic performance, yielding lower AUCs of 0.822 and 0.803, respectively (Supplementary Table 5). Pathophysiologically, these indices reflect distinct yet complementary aspects of ventilatory defects induced by MPs (airflow obstruction vs. physical air-trapping). Retaining all three variables alongside type 2 inflammatory markers (FeNO, blood eosinophils, IgE) provides a more comprehensive, specific screening tool than relying on any single lung function parameter alone. Although this evidence-weighted approach differs from conventional multivariable regression, comparison showed no statistically significant difference in validation performance between our simplified scoring systems and conventional multivariable regression models directly fitted in this validation cohort, for either MP-Detect (AUC: 0.864 vs. 0.852; DeLong test *P* = 0.436) or MP-Severity (AUC: 0.859 vs. 0.932; DeLong test *P* = 0.180). This outcome supports the robustness of using meta-analysis pooled effect sizes as predictor weights to construct generalizable scoring systems.

Furthermore, we successfully translated the complex mathematical equations into a clinically user-friendly simplified scoring system. In the validation cohort, DeLong’s test indicated no statistically significant difference in discriminative performance between the simplified scoring system and its mathematical score counterpart (DeLong test *P* = 0.250). However, a non‑significant DeLong test does not prove statistical equivalence or non‑inferiority; the primary advantage of the simplified scoring system lies in its enhanced clinical usability and ease of bedside application. At a cutoff value of 4.250, the scoring system exhibited a remarkably high specificity of 0.941, which is crucial in clinical screening to substantially reduce misdiagnosis and subsequent unnecessary imaging burden. Decision curve analysis (DCA) further confirmed the net benefit of this scoring system across clinically relevant threshold ranges (approximately 0.1 to 0.9), suggesting a potential role in preliminary risk stratification.

Our study has several limitations. First, the 105‑patient cohort in this study should be considered an internal validation cohort rather than a true external validation set, and its single‑center, retrospective nature with a modest sample size limits the generalizability of our findings. Second, the cutoffs for age, IgE, and BMI were derived from this same cohort, which may contribute to optimistic bias. Although 10‑fold cross‑validation and bootstrap internal validation demonstrated good stability with minimal overfitting (optimism: 0.017 for both MP‑Detect and MP‑Severity in the mathematical model; 0.017 and 0.018 in the simplified model), prospective, multicenter external validation studies are essential to confirm the scoring system’s generalizability before routine clinical implementation. Third, our scoring system is primarily targeting adult asthma patients. Its applicability in children or specific asthma phenotypes requires further investigation. Fourth, we did not explore dynamic changes in the score following therapeutic interventions. Future research should evaluate the scoring system’s utility in predicting responses to biologics and guiding airway clearance therapies. Fifth, sputum mucin protein (MUC5B and MUC5AC) quantification was not performed, future prospective studies integrating sputum mucin biomarkers are warranted.

In conclusion, the MP-Detect Score and MP-Severity Index developed in this study represent preliminary evidence‑weighted clinical scoring tools that may, after prospective external validation, help identify asthma patients at risk of airway mucus plugs and prioritize them for further HRCT evaluation.

## Supplementary Information

Supplementary Material 1.

Supplementary Material 2.

Supplementary Material 3.

Supplementary Material 4.

## Data Availability

The datasets generated during and/or analyzed during the current study are available from the corresponding author upon reasonable request.

## Abbreviations

MPs: Airway mucus plugs
FEV1%: Percentage forced expiratory volume in 1 s
FVC%: Percentage forced vital capacity
FeNO: Fractional exhaled nitric oxide
ROC: Receiver operating characteristic
AUC: Area under the curve
DCA: Decision curve analysis
