# Supplementary material for: A non-invasive simplified scoring system to predict airway mucus plugs in asthma
Source: BMC Pulm Med. 2026 Aug 4;26:512. doi: 10.1186/s12890-026-04560-0 (PMC13721314; doi:10.1186/s12890-026-04560-0)
Supplement: Supplementary file 1 [file 12890_2026_4560_MOESM1_ESM.docx]

**Asthma patients with airway mucus plugs manifest more severe symptoms, airflow limitation, and eosinophilic airway inflammation: a systematic review and meta-analysis**

Zhen Wang ^1,2+^, Tiantian Xiong ^1,2+^, Gongqi Chen ^1,2^, Chunli Huang ^1,2^, Wei Gu ^1,2^, Huiru Jie ^1,2^, Lixia Xia ^3^, Ruchong Chen ^4^, [Yuchen Feng](https://pubmed.ncbi.nlm.nih.gov/?size=200&term=Feng+Y&cauthor_id=35930423) ^1,2*^, Guohua Zhen ^1,2*^

^1^Department of Respiratory and Critical Care Medicine, Tongji Hospital, Tongji Medical College, Huazhong University of Science and Technology, Wuhan, China;

^2^Key Laboratory of Respiratory Diseases, National Health Commission of People’s Republic of China, and National Clinical Research Center for Respiratory Diseases, Wuhan, China;

^3^Key Laboratory of Respiratory Disease of Zhejiang Province, Department of Respiratory and Critical Care Medicine, Second Affiliated Hospital of Zhejiang University School of Medicine, Hangzhou, China;

^4^State Key Laboratory of Respiratory Disease, National Center for Respiratory Medicine, Guangzhou Institute of Respiratory Health, The First Affiliated Hospital of Guangzhou Medical University, Guangzhou, China.

^+^These authors have contributed equally to this work

*Correspondence to:

Guohua Zhen, Department of Respiratory and Critical Care Medicine, Tongji Hospital, Huazhong University of Science and Technology, Wuhan 430030, China; Email: [ghzhen@tjh.tjmu.edu.cn](mailto:ghzhen@tjh.tjmu.edu.cn).

[Yuchen Feng](https://pubmed.ncbi.nlm.nih.gov/?size=200&term=Feng+Y&cauthor_id=35930423), Department of Respiratory and Critical Care Medicine, Tongji Hospital, Huazhong University of Science and Technology, Wuhan 430030, China; Email: fengyc@tjh.tjmu.edu.cn.

**1. Materials and Methods**

**1.1. Literature Search Strategy**

A systematic search was conducted across PubMed, Embase, Cochrane Library, CNKI, and Wanfang databases up to February 16, 2025. Clinical trials registered on ClinicalTrials.gov were also screened. Search terms included: "asthma," "mucus plugs," "mucus score," "mucus obstruction" (English); "哮喘," "黏液栓," "黏液阻塞" (Chinese).

**1.2. Inclusion and Exclusion Criteria**

Inclusion criteria: (1) Asthma diagnosis per Global Initiative for Asthma (GINA) guidelines; (2) Mucus plugs confirmed radiologically by ≥2 thoracic radiologists; (3) Adult asthma patients; (4) Observational or interventional studies reporting clinical outcomes.

Exclusion criteria: (1) Reviews, letters, or conference abstracts; (2) Inaccessible full texts or incomplete data; (3) Duplicate datasets.

**1.3. The criteria for mucus plug scores**

Considering that the cutoff of mucus plug scores vary between the included studies, we analyzed the data of asthma patients with low (0-1～3-3.5) and high mucus plug (>3.5-4) scores. In the study by Gotschke et al., the authors defined the mucus plug score as mild (1-3), moderate (4-11), and severe (12-20). For this study, we analyzed the patients with mild score and severe score as those with low and high mucus plugs, respectively.

**1.4. Data Extraction and Quality Assessment**

Data were independently extracted by two reviewers from eligible studies. Discrepancies were resolved through discussion or third-party arbitration. The following data were extracted from each study: first author, publication year, region, study type, and clinical data such as lung function.

The Newcastle-Ottawa Scale (NOS) was used to assess the quality of case-control and cohort studies. The NOS evaluates three domains: selection (4 items, 4 points), comparability (1 item, 2 points), and outcome (3 items, 3 points), with a total score of 9. Studies scoring ≥6 were considered high-quality (Grade A), while those scoring <6 were low-quality (Grade B). The Cochrane Risk of Bias Tool was used to assess the risk of bias in randomized controlled trials (RCTs), including bias from randomization, deviations from intended interventions, missing outcome data, outcome measurement, and selective reporting. Each aspect was rated as "low risk," "unclear risk," or "high risk." The Agency for Healthcare Research and Quality (AHRQ) criteria were used to evaluate the quality of cross-sectional studies. The AHRQ includes 11 items, with a total score of 11. Studies scoring ≥8 were considered high-quality, while those scoring ≤3 were low-quality.

**1.5. Statistical Analysis**

The data of this study rely on the reported summary data and statistics included in the original studies. RevMan 5.3 software was used for data analysis. Dichotomous data were expressed as odds ratios (OR) with 95% confidence intervals (CI), while continuous data were expressed as mean differences (MD) with 95% CI. Heterogeneity among studies was assessed using the Q test and I² statistic. A fixed-effects model was used when heterogeneity was not significant (P> 0.1, I² ≤ 50%). When heterogeneity was significant (P≤ 0.1, I² > 50%), a random-effects model was used after further analysis of heterogeneity sources.

**2. Results**

**2.1. Characteristics of Included Studies**

A total of 3,055 articles were initially identified (1,466 from PubMed, 1,185 from Embase, 57 from Cochrane, 47 from CNKI, and 221 from Wanfang). After removing 1,147 duplicates, 1,908 unique articles were retained for further assessment. Screening of titles and abstracts resulted in the exclusion of 1,811 irrelevant articles, including 1,288 non-relevant studies, 321 reviews, and 202 case reports. The remaining 97 articles were thoroughly reviewed, and 87 were excluded for the following reasons: events of asthma and mucus plugs were not reported (n = 25), lack of target study data (n = 27), and not grouped according to mucus plugs (n = 35). Finally, 10 articles were included, with 6 only focusing on the impact of airway mucus plugs on asthma, 1 on both mucus plugs and mucus plug scores, and 3 only on mucus plug scores. The study selection process is shown in Figure 1.

Fig 1. Flow chart of the identification of relevant studies

**2.2. Quality Assessment of Included Studies**

The Cochrane Risk of Bias Tool (RoB 2) was used to assess bias in the included RCT, which was deemed high-quality with no significant bias (Figure 2). The NOS scores for cohort studies were all ≥6 (Table 1), and the cross-sectional study scored 8, indicating overall high quality (Table 2).

Fig 2. Risk assessment of the included literature

Table 1. Quality assessment of included literature (NOS score)

| Included literature | Research object selection | Comparability between groups | Result measurement | NOS score | Quality grade |
| --- | --- | --- | --- | --- | --- |
| Tang 2022 | 3 | 2 | 3 | 8 | **A** |
| Mummy 2022 | 3 | 2 | 2 | 7 | **A** |
| Chan 2023 | 3 | 2 | 3 | 8 | **A** |
| Tamura 2022 | 3 | 2 | 3 | 8 | **A** |
| Yang 2024 | 3 | 1 | 3 | 7 | **A** |
| Dunican  2018 | 3 | 2 | 2 | 7 | **A** |
| Audousset  2024 | 3 | 1 | 2 | 6 | **A** |
| Götschke  2025 | 3 | 2 | 3 | 8 | **A** |

Table 2. Quality assessment of included literature (AHRQ score)

| Included literature | Clear source of data | The inclusion and exclusion criteria for cases and controls were listed | Give the time stage of patient identification | Continuity of subjects | Subjective factors of evaluators cover up other aspects of research objects | Describes the assessment to ensure quality | Explain the reasons for excluding patients from the analysis | Describe measures to evaluate or control confounding factors | Explain how to handle missing data | Patient response rates and completeness of data collection were summarized | Identify the percentage of expected patients with incomplete data or follow-up results | AHRQ score | Quality grade |
| --- | --- | --- | --- | --- | --- | --- | --- | --- | --- | --- | --- | --- | --- |
| Liu. 2023 | **√** | **√** | **√** | **√** | **-** | **√** | **√** | **√** | **×** | **√** | **×** | **8** | **high** |

**3. Meta analysis of the clinical characteristics of asthma patients with and without mucus plug**

**3.1. Asthma patients with mucus plugs are older and more likely to have severe asthma**

Five studies analyzed the relationship between the presence of mucus plugs in asthma and age. Meta analysis revealed that asthma patients with mucus plugs were older. There was no heterogeneity among the studies (I² = 0%, *P* = 0.70), and a fixed-effects model yielded an MD of 6.42 (95% CI: 3.67–9.18, *P*<0.0001), indicating a statistically significant difference (Figure 3a). Four studies showed that mucus plugs were more common in severe asthma patients, with no heterogeneity (I² = 0%, *P* = 0.74) and statistical significance (OR = 4.19, 95% CI: 2.32–7.59, *P*<0.0001) (Figure 3b). This suggests that asthma patients with mucus plugs are older and more likely to have severe asthma.

Fig 3. Meta-analysis of the relationship between asthma with mucus plugs and age and severe asthma

**3.2. Airflow limitation is more severe in asthma patients with mucus plugs**

FEV_1_%, FVC%, and FEV_1_/FVC% were used to assess airflow limitation. For FEV_1_%, significant heterogeneity was observed among six studies (I² = 79%, *P* < 0.0001) (Supplementary Figure 1a). Subgroup analysis by study type revealed one cross-sectional study and one RCT, with the remaining four being cohort studies. Heterogeneity decreased significantly (I² = 40%, *P* = 0.17), suggesting study type as the source of heterogeneity. A random-effects model showed that FEV_1_% was lower in asthma patients with mucus plugs compared to those without mucus plugs (MD = -18.54, 95% CI: -24.37 to -12.71, *P* < 0.0001) (Figure 4a). Similarly, FVC% was lower in these patients (MD = -8.78, 95% CI: -12.07 – -5.50, *P* < 0.0001) (Figure 4b). For FEV_1_/FVC%, significant heterogeneity was observed among five studies (I² = 63%, *P* = 0.03) (Supplementary Figure 1b). Subgroup analysis by ethnicity revealed two studies in Asians and three in Caucasians, with reduced heterogeneity (I² = 12%, *P* = 0.32). A random-effects model showed that FEV_1_/FVC% was lower in Caucasians with mucus plugs (MD = -11.46, 95% CI: -13.91 – -9.01, *P* < 0.0001) (Figure 4c), while the two Asian studies also showed statistical significance (Supplementary Figure 1c). This suggests that airflow limitation is more severe in asthma patients with mucus plugs.

Fig 4. Meta-analysis of the relationship between asthma with or without mucus plug and lung function

**3.3. Asthma patients with mucus plugs have more severe airway inflammation**

The mean difference in sputum eosinophil percentage between asthma patients with and without mucus plugs was 5.09, with statistical significance (95% CI: 2.90–7.28; *P* < 0.0001; 3 studies, 219 participants; Figure 5a). There was no heterogeneity among the studies (I² = 25%, *P*=0.26). Similarly, peripheral blood eosinophil count (MD = 125.09, 95% CI: 83.84–166.35, *P* < 0.0001), sputum neutrophil percentage (SMD = -0.42, 95% CI: -0.70 – -0.14, *P* = 0.004), and IgE levels (SMD = 0.37, 95% CI: 0.18–0.55, *P* < 0.0001) were higher in asthma patients with mucus plugs, with statistical significance (Figures 5b, 5c, 5d). Five studies reported higher FeNO levels in asthma patients with mucus plugs (SMD = 0.70, 95% CI: 0.51–0.90, *P* < 0.0001) (Figure 5e). This suggests that asthma patients with mucus plugs have more severe airway inflammation.

Fig 5. Meta-analysis of the relationship between mucus plug and airway inflammation in asthma

**3.4.** **Asthma patients with mucus plugs may be more likely to have acute exacerbations**

A total of three literatures studied the relationship between airway mucus plugs and acute exacerbation of asthma. We found that it was statistically significant (MD=1.11, 95%CI=0.83-1.39, *P* < 0.0001), but the heterogeneity was large (I^2^=95%, *P* < 0.0001). The heterogeneity was still significant when the random-effects model was adjusted for meta-analysis (I^2^=95%, *P* < 0.0001) (Figures 6a, 6b). Considering the different definitions of acute exacerbations in the article, in the study by Professor Chan et al., it was defined as "Oral corticosteroid exacerbations" (Table 3). After exclusion, a meta-analysis of the remaining two studies showed statistical significance (*P* = 0.001) (Figure 6c). This suggest that asthma patients with mucus plugs may be more likely to have acute exacerbations.

Fig 6. Meta-analysis of the relationship between asthma with mucus plug and acute exacerbation

Table 3. Comparison of sources of literature heterogeneity

| Research | **Population characteristics** | **Specific intervention** | **Definition of acute exacerbation** |
| --- | --- | --- | --- |
| Chan 2023 | Adult patients with asthma | None | Acute exacerbation occurred with oral corticosteroids |
| Nordenmark 2023 | Adult patients with asthma | Tezepelumab | Not clearly defined |
| Tamura 2022 | Adult patients with asthma | None | Not clearly defined |

**4. Meta analysis of the clinical characteristics of asthma patients with high and low mucus plug scores**

**4.1. Asthma patients with high mucus plug scores are older and have higher peripheral blood eosinophil counts and FeNO**

Four studies examined the relationship between mucus plug scores and age, with significant heterogeneity (I² = 71%, *P* = 0.02) (Supplementary Figure 2a). Subgroup analysis by study type revealed one RCT and three cohort studies, with reduced heterogeneity (I² = 0%, *P* = 0.60). A random-effects model showed that patients with higher mucus plug scores were older than patients with low mucus plug scores. (MD = 5.90, 95% CI: 1.77–10.03, *P* = 0.005) (Figure 7a), while the RCT showed no statistical significance (*P* = 0.05). Patients with higher mucus plug scores also had higher peripheral blood eosinophil counts (MD = 179.64, 95% CI: 98.38–260.91, *P* < 0.0001) and FeNO levels (SMD = 0.56, 95% CI: 0.29–0.84, *P* < 0.0001), compared with low mucus plug scores (Figure 7b, c). This suggests that asthma patients with high mucus plug scores are older and have higher peripheral blood eosinophil counts and FeNO.

Fig 7. Meta-analysis of the relationship between mucus plug score and age, blood eosinophil count and FeNO

**4.2.** **Asthma with high mucus plug score have lower BMI and FEV_1_%**

Four studies described the relationship between mucus plug scores and BMI (Figure 8a). Patients with higher mucus plug scores had lower BMI, , with no heterogeneity (*P* = 0.69, I² = 0%) and statistical significance (95% CI: -4.38 – -0.74, *P* = 0.006). For FEV_1_%, heterogeneity was low (*P* = 0.31, I² = 15%), and a fixed-effects model yielded an SMD of -0.76 (95% CI: -1.04 – -0.48, *P* < 0.0001), indicating statistical significance(Figure 8b). This suggests that asthma with high mucus plug score had lower BMI and FEV_1_%.

Fig 8. Meta-analysis of the relationship between mucus plugs score and BMI, FEV_1_%

Among the 15 indicators examined for the impact of mucus plugs on asthma, 10 showed statistical significance: age, severe asthma, FEV_1_%, FVC%, FEV_1_/FVC%, IgE, FeNO, sputum eosinophil percentage, sputum neutrophil percentage, and peripheral blood eosinophil count. The remaining five indicators showed no statistical significance (Table 4; Supplementary Figure 3). Among the six indicators examined for the impact of mucus plug scores on asthma, five showed statistical significance: age, BMI, FEV_1_%, FeNO, and peripheral blood eosinophil count, while gender (female) showed no statistical significance (Table 5; Supplementary Figure 2b).

Table 4. Results of meta-analysis of clinical characteristics of asthma complicated with mucous plug

| Clinical features | Literature quantity | Heterogeneity test | | Analysis model | OR/MD/  SMD | 95%CI | *P* |
| --- | --- | --- | --- | --- | --- | --- | --- |
|  |  | I²(%) | P |  |  |  |  |
| Gender（Female） | 6 | 32 | 0.20 | Fixed | 0.89 | 0.62～1.29 | 0.55 |
| Smoking history | 3 | 0 | 46 | Fixed | 1.26 | 0.66～2.43 | 0.48 |
| Age | 5 | 0 | 0.7 | Fixed | 6.42 | 3.67～9.18 | <0.0001 |
| BMI | 6 | 38 | 0.15 | Fixed | 0.43 | -0.25～1.12 | 0.22 |
| Severe asthma | 4 | 0 | 0.74 | Fixed | 4.19 | 2.32～7.59 | <0.0001 |
| FEV_1_% | 4 | 40 | 0.17 | Random | -18.54 | -24.37～-12.71 | <0.0001 |
| FVC% | 5 | 8 | 0.36 | Fixed | -8.78 | -12.07～-5.5 | <0.0001 |
| FEV_1_/FVC% | 3 | 12 | 0.32 | Random | -11.46 | -13.91～-9.01 | <0.0001 |
| FeNO | 5 | 35 | 0.19 | Fixed | 0.7 | 0.51～0.90 | <0.0001 |
| IgE | 5 | 0 | 0.45 | Fixed | 0.37 | 0.18～0.55 | <0.0001 |
| Sputum eosinophil % | 3 | 25 | 0.26 | Fixed | 5.09 | 2.90～7.28 | <0.0001 |
| Sputum neutrophil % | 3 | 21 | 0.28 | Fixed | -0.42 | -0.70～-0.14 | 0.004 |
| Blood eosinophil count | 5 | 0 | 0.90 | Fixed | 125.09 | 83.84～166.35 | <0.0001 |
| Blood neutrophil count | 3 | 0 | 0.89 | Fixed | -203.29 | -516.67～110.09 | 0.20 |
| Acute Exacerbations | 3 | 95 | <0.0001 | Random | 1.2 | -0.20～2.60 | 0.09 |

Table 5. Results of meta-analysis of clinical characteristics of asthma combined with mucus plug score

| Clinical features | Literature quantity | Heterogeneity test | | Analysis model | OR/MD/  SMD | 95%CI | *P* |
| --- | --- | --- | --- | --- | --- | --- | --- |
|  |  | I²(%) | P |  |  |  |  |
| Age | 3 | 0 | 0.60 | Random | 5.90 | 1.77～10.03 | 0.005 |
| Gender (Female) | 4 | 0 | 0.86 | Fixed | 0.75 | 0.44～1.28 | 0.29 |
| BMI | 4 | 0 | 0.69 | Fixed | -2.56 | -4.38～-0.74 | 0.006 |
| FEV_1_% | 4 | 15 | 0.31 | Fixed | -0.76 | -1.04～-0.48 | <0.0001 |
| FeNO | 4 | 36 | 0.20 | Fixed | 0.56 | 0.29～0.84 | <0.0001 |
| Blood eosinophil count | 3 | 0 | 0.87 | Fixed | 179.64 | 98.38～260.91 | <0.0001 |

**5. Publication Bias**

Egger's test was used to assess publication bias for studies with ≥3 included articles. Results showed *P* > 0.05 and 95% CI including 0, indicating no significant publication bias (Table 6、7).

Table 6. Publication bias of study indicators of asthma combined with mucus plugs (Egger's)

| Research Indicators | *P*-value |
| --- | --- |
| Gender（Female） | 0.210 |
| Smoking history | 0.80 |
| Severe asthma | 0.119 |
| Age | 0.452 |
| BMI | 0.775 |
| FEV1% | 0.623 |
| FVC% | 0.774 |
| FEV1/FVC% | 0.885 |
| FENO | 0.278 |
| IgE | 0.068 |
| Sputum eosinophil % | 0.485 |
| Blood eosinophil count | 0.785 |
| Sputum neutrophil % | 0.369 |
| Blood neutrophil count | 0.262 |
| Acute Exacerbations | 0.849 |

Table 7. Publication bias of study indicators of asthma combined with mucus plug score (Egger's)

| Research Indicators | *P*-value |
| --- | --- |
| Gender（Female） | 0.794 |
| Age | 0.755 |
| BMI | 0.104 |
| FEV1% | 0.822 |
| Blood eosinophil count | 0.870 |
| FENO | 0.141 |
