# Supplementary material for: A non-invasive simplified scoring system to predict airway mucus plugs in asthma
Source: BMC Pulm Med. 2026 Aug 4;26:512. doi: 10.1186/s12890-026-04560-0 (PMC13721314; doi:10.1186/s12890-026-04560-0)
Supplement: Supplementary file 2 [file 12890_2026_4560_MOESM2_ESM.docx]

Fig 1. Meta-analysis of the relationship between asthma with mucus plugs and lung function

Fig 2. Meta-analysis of mucus plug score and related indicators

Fig 3. Meta-analysis of asthma with mucus plug and related indicators
