# Supplementary material for: A non-invasive simplified scoring system to predict airway mucus plugs in asthma
Source: BMC Pulm Med. 2026 Aug 4;26:512. doi: 10.1186/s12890-026-04560-0 (PMC13721314; doi:10.1186/s12890-026-04560-0)
Supplement: Supplementary file 4 [file 12890_2026_4560_MOESM4_ESM.docx]

**Supplementary** table 1. Cut-off values for risk factors of mucus plug formation

| **Mucus Plugs (Presence vs Absence)** | | | |
| --- | --- | --- | --- |
| Risk factors | Cut-off | Predictive direction | AUC |
| Age | > 52.0 | Positive correlation | 0.833 |
| FEV_1_% | < 73.1 | Negative correlation | 0.798 |
| FEV_1_/FVC% | < 64.91 | Negative correlation | 0.798 |
| blood eosinophil count (*10^9/L) | > 0.35 | Positive correlation | 0.699 |
| FVC% | < 102.7 | Negative correlation | 0.719 |
| FeNO (ppb) | > 35.0 | Positive correlation | 0.539 |
| IgE (IU/mL) | > 611.4 | Positive correlation | 0.522 |
| **Mucus Plugs (High vs Low Score)** | | | |
| Age | > 52.0 | Positive correlation | 0.948 |
| FEV_1_% | < 77.9 | Negative correlation | 0.772 |
| blood eosinophil count (*10^9/L) | > 0.35 | Positive correlation | 0.695 |
| FeNO | > 35.0 | Positive correlation | 0.578 |
| BMI(kg/m²) | > 19.53 | Positive correlation | 0.525 |

**Supplementary** table 2. Normalization constants (means and standard deviations) for Z-score transformation.

| Variable | Mean | SD |
| --- | --- | --- |
| Age | 51.73 | 11.48 |
| FEV₁% | 75.29 | 24.20 |
| FVC% | 99.66 | 19.76 |
| FEV₁/FVC% | 61.74 | 12.83 |
| FeNO | 44.58 | 39.14 |
| Blood eosinophil count | 0.42 | 0.46 |
| IgE | 416.72 | 497.34 |

**Supplementary** table 3. Internal validation performance of the mathematical equation‑based score and simplified scoring system

|  | Outcome | Apparent AUC | Bootstrap‑corrected AUC | Optimism | 95% CI | 10‑fold CV (mean ± SD) | Brier Score | Calibration Slope |
| --- | --- | --- | --- | --- | --- | --- | --- | --- |
| Mathematical | MP‑Detect | 0.839 | **0.822** | 0.017 | 0.755–0.923 | 0.825 ± 0.075 | 0.153 | 1.039 |
| Mathematical | MP‑Severity | 0.831 | **0.814** | 0.017 | 0.741–0.921 | 0.818 ± 0.084 | 0.119 | 0.971 |
| Simplified | MP‑Detect | 0.864 | 0.847 | 0.017 | 0.768–0.926 | 0.846 ± 0.069 | 0.142 | 1.021 |
| Simplified | MP‑Severity | 0.859 | 0.841 | 0.018 | 0.759–0.923 | 0.842 ± 0.088 | 0.158 | 0.984 |

**Supplementary** table 4. Comparison of predictive performance between multivariable logistic regression and the simplified scoring system

| Outcome | Model | AUC | 95% CI | DeLong test (vs. Simplified) |
| --- | --- | --- | --- | --- |
| **MP-Detect** | Multivariable logistic regression | 0.852 | 0.752–0.918 | - |
|  | Simplified scoring system | 0.864 | 0.785–0.943 | Z = 0.78, P = 0.436 |
| **MP-Severity** | Multivariable logistic regression | 0.932 | 0.864–0.999 | - |
|  | Simplified scoring system | 0.859 | 0.777–0.941 | Z = 1.341, P = 0.180 |

### **Supplementary table 5. Collinearity Diagnostics and Sensitivity Analysis of the Lung Function Variables in the Validation Cohort (**n=105**)**

|  | Variables Included | VIF | AUC (95% CI) | Sensitivity (%) | Specificity (%) |
| --- | --- | --- | --- | --- | --- |
| Mathematical equation‑based score (MP-Detect Score) | Age, FeNO, Blood eosinophil count, IgE, **FEV_1_**%**, **FVC%**, FEV_1_**/FVC%**** | 12.86 (FEV_1_%) 7.95 (FVC%) 6.35 (FEV_1_/FVC%) | 0.839 (0.755–0.923) | 77.8% | 87.5% |
| **Restricted system** | Age, FeNO, Blood eosinophil count, IgE, **FEV_1_**/FVC%**** | N/A | 0.812 (0.721–0.903) | 74.6% | 82.4% |
| **Restricted system** | Age, FeNO, Blood eosinophil count, IgE, **FEV_1_**%**** | N/A | **0.822** (0.735–0.909) | **81.0%** | **78.4%** |
| **Restricted system** | Age, FeNO, Blood eosinophil count, IgE, ****FVC%**** | N/A | **0.803** (0.710–0.896) | **76.2%** | **80.4%** |
